# Novel staging for gastric neuroendocrine neoplasms by incorporating the WHO grading into the TNM staging system

**DOI:** 10.1002/cam4.5437

**Published:** 2022-11-16

**Authors:** Rihong Zhang, Yu Guo, Youliang Wang, Li Hu, Cheng Fang, Yujie Yang, Xianqi Yang, Luohai Chen, Jie Chen, Wei Wang, Xiaowei Sun

**Affiliations:** ^1^ Department of Gastric Surgery, State Key Laboratory of Oncology in South China, Collaborative Innovation Center for Cancer Medicine Sun Yat‐sen University Cancer Center Guangzhou Guangdong P. R. China; ^2^ Department of Gastroenterology The First Affiliated Hospital of Sun Yat‐sen University Guangzhou Guangdong P. R. China

**Keywords:** American joint committee on cancer, gastric neuroendocrine neoplasms, surveillance, epidemiology and end result, TNM, WHO grade

## Abstract

**Background:**

The 8th tumor‐node‐metastasis (TNM) classification of the American Joint Committee on Cancer (AJCC) can be used to estimate the prognosis of gastric neuroendocrine tumor (gNET) and gastric neuroendocrine carcinoma (gNEC) patients but not gastric neuroendocrine neoplasms (gNENs).

**Methods:**

First, in the SEER (training) dataset, a TNMG system was built by combining the WHO G grade (G1‐4; NEC grouped into G4) with the 8th AJCC T (T1‐4), N (N0‐1), and M (M0‐1) stage, which was then validated in a Chinese (validation) cohort.

**Results:**

In all, 2245 gNENs cases from the training dataset and 280 cases from the validation dataset were eligible. The T stage, M stage, and G grade were independent prognostic factors for OS in both datasets (all *p <* 0.05). The TNMG staging system demonstrated better C‐index for predicting OS than the 8th AJCC TNM staging system in both the training (0.87, 95%CI: 0.86–0.88 vs. 0.79, 95%CI: 0.77–0.81) and validation (0.77, 95%CI: 0.73–0.80 vs. 0.75, 95%CI: 0.71–0.79) datasets. The AUC of the 3‐year OS for the TNMG staging system was 0.936 and 0.817 in the SEER and validation dataset, respectively; higher than those of the 8th AJCC system (vs. 0.843 and 0.779, respectively). DCA revealed that compared with the 8th AJCC TNM staging system, the TNMG staging system demonstrated superior net prognostic benefit in both the training and validation datasets.

**Conclusions:**

The proposed TNMG staging system could more accurately predict the 3‐ and 5‐year OS rate of gNENs patients than the 8th AJCC TNM staging system.

## Graphical abstract

The proposed TNMG staging system for gastric neuroendocrine neoplasms was successfully validated in a two‐center dataset of a different ethnicity than the SEER training dataset. This novel staging system could more accurately prognosticate the 3‐ and 5‐year OS rate than the 8th AJCC TNM staging system.

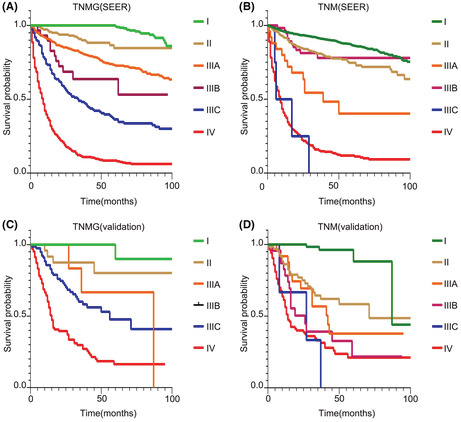

## 1 INTRODUCTION

Gastric neuroendocrine neoplasms (gNENs) arise from the subepithelial, histamine‐secreting, enterochromaffin‐like cells.^1^ With deeper understanding on gNENs and improvements in diagnostic techniques, the worldwide incidence of gNENs has increased over the past few decades.^2^, ^3^, ^4^ The World Health Organization (WHO) proposed a new classification in 2019 that divided gNENs into gastric neuroendocrine tumors (gNETs) and gastric neuroendocrine carcinoma (gNEC). Besides, the WHO grade (G grade) of neuroendocrine neoplasms (NENs) was reclassified as NET‐G1, NET‐G2, NET‐G3, and NEC (grouped into G4 in the article), based on Ki‐67 index, mitotic rate and differentiation,^5^ but is not widely clinically used.

At present, the staging criteria of the 8th American Joint Committee on Cancer (AJCC) tumor‐node‐metastasis (TNM) staging system for gastric neuroendocrine tumor (gNET) and gastric neuroendocrine carcinoma (gNEC) are different. The 8th AJCC staging system for gastric NETs (NET‐AJCC) can only be applied to gNETs, while the 8th AJCC staging system for gastric cancer (GC‐AJCC) is also used for predicting the survival of gNEC patients, which has led to inconvenience for clinicians when making therapeutic decisions for patients with gNENs.^6^

Though the AJCC TNM staging system and G grade were used widely to help guide the management for gNENs,^7^, ^8^ some inadequacies were observed when implementing the 8th AJCC TNM staging system. One study on primary pancreatic neuroendocrine tumors (pNETs) found that when using the 8th AJCC TNM staging system, the long‐term OS of patients of stage I and IIA (*p* = 0.988), IIB and IIIA (*p* = 0.462), and overall stage I and II (*p* = 0.188) were similar in both the SEER and a multi‐institutional database.^9^ Wang et al. analyzed the features of 644 patients with pancreatic neuroendocrine carcinoma (pNEC) and found no statistical significance in hazard ratio (HR) between stage I and II disease in multivariate analyses (stage I, served as reference, vs. stage II: HR, 0.74; *p* = 0.32).^10^ One study on gNENs found that three‐year OS were 92.6%, 89.8%, 93.5%, and 40.9% for gNET patients at the 8th AJCC I, II, III, and IV staging, respectively. At the same time, there was no statistical difference in the prognosis of gNET patients at the 8th AJCC I, II, and III staging (*p* > 0.05).^11^ Another relevant study on gNEC found that compared to the 8th AJCC TNM staging system, the 6th AJCC staging system was more suitable for gNEC.^12^ These studies illustrated that the 8th AJCC TNM staging system could not stratify patients with gNENs. Since the 8th AJCC TNM staging system has limitations in stratifying patients with gNENs, and at the same time, gNENs with different tumor grades and characteristics have different prognoses and treatments,^1^, ^13^ a more robust staging system is needed to accurately predict the prognosis of gNENs patients and assist clinical therapeutic decisions.^14^

In this study, we propose a novel staging system (TNMG) by combining the T stage, N stage, M stage, and G grade using the data of patients from the Surveillance, Epidemiology and End Result (SEER) database and validated the model in a dataset of different ethnicity. The clinical performance of the proposed TNMG system and 8th AJCC staging system was also compared.

## 2 MATERIALS AND METHODS

### 2.1 SEER database patients' retrieval and eligibility criteria

The SEER database was inspected for patients pathologically diagnosed as gNENs and treated from 2004 to 2018. The International Classification of Diseases for Oncology (ICD‐O‐3) was used to identify gNENs cases. The following ICD‐O‐3 codes for histological type were used: large cell carcinoma (8012), large cell neuroendocrine carcinoma (8013), small cell carcinoma (8041), carcinoid tumor (8240), mixed adenoneuroendocrine carcinoma (8244), neuroendocrine carcinoma (8246). The ‘Site recode ICD‐O‐3/WHO 2008’ data were set as ‘stomach’ to filter cases by tumor location. Year of diagnosis was set from 2004 to 2018. The TNM staging data were retrieved based on the following codes: derived EOD 2018 stage group (2018+), derived 7th AJCC TNM stage (2010–2015), derived SEER combined TNM stage group (2016–2017), derived 6th AJCC TNM stage (2004–2015). Survival information were retrieved using the terms ‘vital status recode’ and ‘survival months’. Further, the following codes were used to screen out the included patients: pathologically diagnosed as gNENs, and complete follow‐up and outcomes data. Patients from the SEER cohort were labeled as the training cohort.

### 2.2 Chinese patients' retrieval, patient stratification, and study endpoints

The data from gNENs patients treated at the Sun Yat‐sen University Cancer Center (SYSUCC) and The First Affiliated Hospital of Sun Yat‐sen University from July 2008 to August 2018 were retrieved and analyzed. Patients satisfying the following criteria were included: 1. pathologically diagnosed as gNENs; 2. had complete information on T stage, N stage, M stage, and G grade; 3. had complete follow‐up data. Patients with the following criteria were excluded: 1. had metastasis of NENs, and; 2. had other tumors besides gNENs. This cohort of patients was labeled as the external validation cohort.

A flow diagram of the selection process is presented in Figure S1. The study survival endpoint was overall survival (OS), calculated from the date of initial diagnosis to the date of last contact or death of any cause. The last date of follow‐up was August 31, 2018. All patients provided informed consent for the anonymous use of their data for scientific purposes prior to treatment. This study protocol was approved by the institutional review boards of each participating institution.

### 2.3 Statistical analysis

Seven clinicopathological variables, including age, sex, primary gastric tumor site, G grade, T stage, N stage, and M stage that could affect the prognosis of gNENs patients, were included in this study. Survival curves were drawn using the Kaplan–Meier method and univariate analysis was performed using the Log‐rank test. Statistically significant variables (*p <* 0.05) in univariate analysis were included in multivariate analysis. Multivariate analysis was performed using the Cox proportional hazards regression with hazard ratios (HRs) and their corresponding 95% confidence intervals (CIs) were computed. Each factor of the T stage (T1–T4), N stage (N0, N1), M stage (M0, M1), and G grade (G1–G4) were combined, after which 29 categories were obtained. Each category had a survival curve. Then, we established the TNMG staging system according to these survival curves. Two rules were then used to create rational stages for the TNMG system: (1) survival curves for specific disease stages that were overlapping could be combined into a single stage; (2) adjacent curves, without statistically significant difference, could be combined into a single stage.^15^, ^16^, ^17^ Finally, the TNMG staging system was established using the SEER dataset and was then externally validated using the Chinese external validation dataset. The Harrell's c‐statistics (C‐index) was used to calculate the prognostic accuracy of the TNMG and 8th AJCC TNM staging systems. The AUCs, DCA and calibration curves were used to evaluate and compare the clinical performance of different staging systems in predicting OS at various time points. All statistical analyses were performed using the Statistical Package For The Social Sciences (SPSS; version 25.0) and R software (R version 4.0.5). *p <* 0.05 was used to determine statistical significance.

## 3 RESULTS

### 3.1 Identification of independent factors

A total of 2245 gNENs cases from the SEER dataset and 280 cases from the Chinese validation dataset were eligible for this study. Univariate analysis identified age, primary site, G grade, T stage, N stage and M stage as significantly associated with the prognosis of patients in the two datasets, except for gender, which was only associated with prognosis in the training dataset (*p <* 0.001; Table 1). There were only a small number of patients with gNET‐G3 in both the training and validation dataset (training: *n* = 37/2245; 1.65%; validation: *n* = 2/280; 0.71%). Multivariate analysis showed that G grade, T stage, M stage were independent prognostic factors in both datasets (Table 2). The HR values of G grade and M stage were the highest, 2.270 (95% CI: 2.027–2.542) and 3.915 (95% CI: 3.274–4.681), respectively.

**TABLE 1:** Clinicopathologic variables and univariate analysis of the gNENs patients

Variables	SEER dataset			Validation dataset		
	Cases (%)	*p* value	HR (95%CI)	Cases (%)	*p* value	HR (95%CI)
Age		<0.001			<0.001	
<60	867 (38.62)		Reference	153 (54.64)		Reference
≥60	1378 (61.38)		2.338 (1.966–2.780)	127 (45.36)		2.055 (1.420–2.976)
Sex		<0.001			0.101	
Female	1011 (45.03)		Reference	192 (68.57)		Reference
Male	1234 (54.97)		2.690 (2.304–3.139)	88 (31.43)		—
Tumor location		<0.001			<0.001	
Upper	540 (24.05)		Reference	111 (39.64)		Reference
Middle	1403 (62.49)		0.326 (0.277–0.384)	80 (28.57)		0.365 (0.217–0.613)
Lower	213 (9.49)		0.661 (0.518–0.844)	43 (15.36)		0.641 (0.368–1.117)
Other	89 (3.97)		0.831 (0.601–1.149)	46 (16.43)		0.834 (0.515–1.353)
G grade		<0.001			<0.001	
G1	924 (41.16)		Reference	60 (21.43)		Reference
G2	200 (8.91)		6.606 (3.705–11.778)	43 (15.36)		17.943 (2.333–137.999)
G3	37 (1.65)		30.019 (16.018–56.258)	2 (0.71)		—
G4	1084 (48.28)		36.686 (23.497–57.278)	175 (62.50)		53.990 (7.529–387.135)
T stage (8th)		<0.001			<0.001	
T1	1575 (70.15)		Reference	74 (26.43)		Reference
T2	376 (16.75)		1.071 (0.868–1.322)	43 (15.36)		18.406 (4.267–79.395)
T3	147 (6.55)		2.641 (2.091–3.337)	87 (31.07)		26.404 (6.406–108.839)
T4	147 (6.55)		5.074 (4.104–6.272)	76 (27.14)		39.417 (9.579–162.197)
N stage (8th)		<0.001			<0.001	
N0	1734 (77.24)		Reference	111 (39.64)		Reference
N1	354 (15.77)		4.927 (4.170–5.821)	152 (54.29)		4.218 (2.587–6.877)
N2	31 (1.38)		5.303 (3.498–8.039)	7 (2.50)		2.569 (0.762–8.668)
N3	12 (0.53)		12.502 (6.998–22.332)	10 (3.57)		12.928 (5.837–28.633)
NA	114 (5.08)		—	—		—
M stage		<0.001			<0.001	
M0	1797 (80.04)		Reference	195 (69.64)		Reference
M1	448 (19.96)		10.912 (9.338–12.751)	85 (30.36)		3.663 (2.539–5.258)

Abbreviations: CI, confidence interval; gNENs, gastric neuroendocrine neoplasms; HR, hazard ratio; NA, not applicable; no., number; SEER, Surveillance, Epidemiology, and End Result.

**TABLE 2:** Multivariate survival analyses of the training and external validation cohorts of gNENs patients

Variables	SEER dataset			Validation dataset		
	*p* value	HR	95% CI	*p* value	HR	95%CI
Age	<0.001	1.911	1.605–2.277	>0.05	—	—
Sex	<0.001	1.487	1.267–1.744	>0.05	—	—
Location	0.022	0.888	0.807–0.978	>0.05	—	—
G grade	<0.001	2.270	2.027–2.542	<0.001	2.335	1.619–3.370
T stage	<0.001	1.198	1.119–1.282	0.024	1.358	1.028–1.793
N stage	<0.001	1.144	1.076–1.215	>0.05	—	—
M stage	<0.001	3.915	3.274–4.681	<0.001	2.675	1.756–4.074

Abbreviations: CI, confidence interval; gNENs, gastric neuroendocrine neoplasms; HR, hazard ratio; SEER, Surveillance, Epidemiology, and End Result.

### 3.2 The TNMG staging system

**FIGURE 1:**
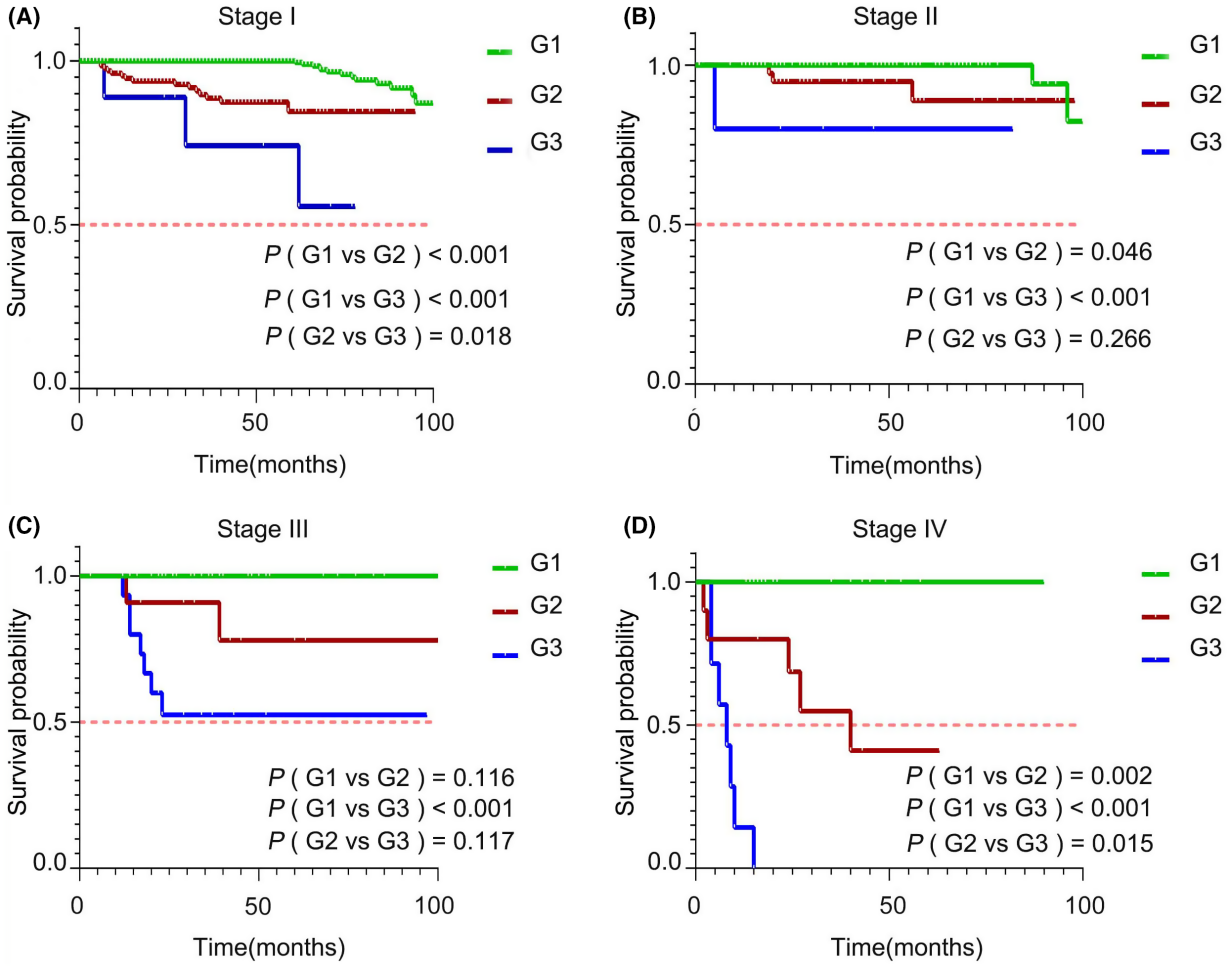
gNET patients' prognostic curves of different G grade in the same TNM stage. Prognostic curves of gNET patients with different G grade were shown in the 8th AJCC TNM stage I, *p* < 0.05 (A). Prognostic curves of gNET patients with different G grade were shown in the 8th AJCC TNM stage II. Prognosis of gNET patients (G1 vs. G2; G1 vs. G3) showed statistical difference (*p* < 0.05), but prognosis of patients (G2 vs. G3) did not (*p* = 0.266) (B). Prognostic curves of gNET patients with different G grade were shown in the 8th AJCC TNM stage III. Prognosis of gNET patients (G1 vs. G3) showed statistical difference (*p* < 0.05) (C). Prognostic curves of gNET patients with different G grade were shown in the 8th AJCC TNM stage IV, *p* < 0.05 (D). gNET, gastric neuroendocrine tumor; TNM, tumor–node–metastasis.

G grade is an important indicator for predicting the prognosis of gNENs patients. Before restaging, we investigated whether patients with different G grades from the same TNM stage had different prognoses. Figure 1 indicates that irrespective of the different TNM stages (I, II, III, or IV), the prognosis of patients of the same stage was statistically different when considering G grade (*p <* 0.05); suggesting that the 8th AJCC TNM staging system is not an optimal clinical model to accurately predict the prognosis of gNENs patients, and inclusion of the G grade could be clinically meaningful.

Further assessment showed that the N stage was an independent prognostic factor in the SEER dataset but not in the validation dataset. Considering the SEER dataset comprised of a larger sample size (*n* = 2245), compared to the validation dataset (*n* = 280), and several previous studies had revealed that the N stage was an independent prognostic factor in gNENs,^11^, ^18^, ^19^, ^20^ the N stage was taken into consideration when constructing the TNMG staging system.

#### 3.2.1 Stage I

**FIGURE 2:**
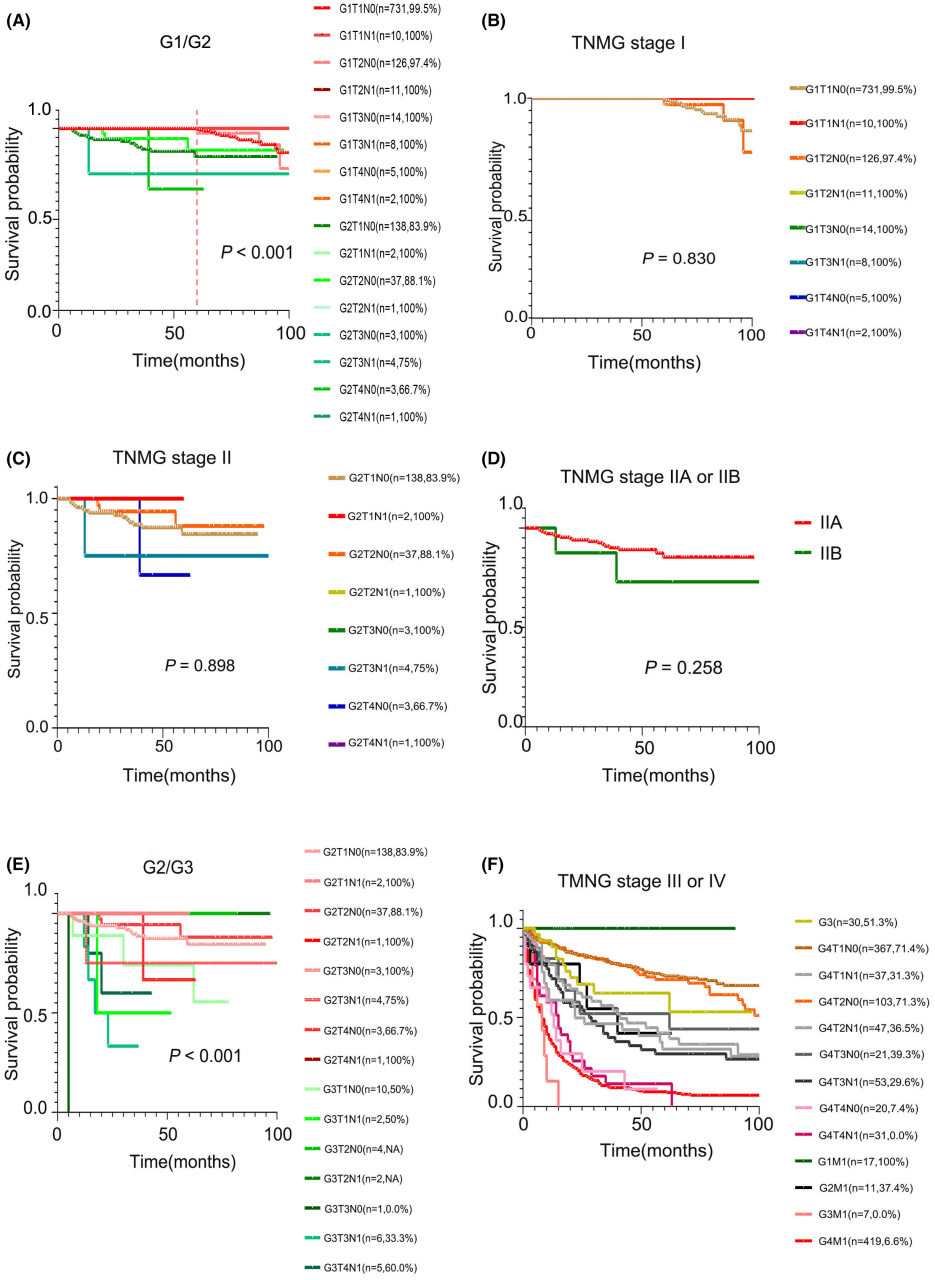
The restaging procedure of TNMG staging system in the SEER dataset. Patients with gNET G1 and G2 were analyzed together (A) and gNET G2 showed worse prognosis compared with gNET G1, *p* < 0.001 (S2A). Number and 5‐year OS rate of patients were shown in every subgroup. And 8 prognostic curves of patients with gNET G1 were included in TNMG stage I, *p* = 0.830 (B). All patients with gNET G2 were classified to TNMG stage II, *p* = 0.898 (C). Two prognostic curves of TNMG stage IIA and IIB showed no statistical difference, *p* = 0.258 (D). Patients with gNET G2 and G3 were analyzed together (E) and gNET G3 showed worse prognosis compared with gNET G2, *p* < 0.001 (S2B). Patients with gNET G3 and gNEC were analyzed together in TNMG stage III and IV (F). gNEC, gastric neuroendocrine carcinoma; gNET, gastric neuroendocrine tumor; TNMG, T stage, N stage, M stage and G grade.

Figure 2A (detailed subgroup comparison G1 vs. G2) and Figure S2A (overall group comparison for G1 vs. G2) both show the comparison of gNET‐G1 versus G2. As could be seen on the figure, the *p* value was <0.001, therefore, G1 and G2 could be separately classified. The TNMG stage I was represented by 8 survival curves, from G1T1N0 to G1T4N1. All gNET‐G1 patients were included in this stage (*p* = 0.830). Among them, G1T1N0, G1T1N1, and G1T2N0 patients accounted for the majority of the patients. Only three survival curves of the larger proportion subgroup are shown in Figure 2B because the other 5 survival curves of the smaller proportion subgroup overlapped and their 5‐year OS rates were almost 100%, similar to G1T1N0, G1T1N1, and G1T2N0 (Figure 2A,B).

#### 3.2.2 Stage II

The TNMG stage II was represented by 8 survival curves, from G2T1N0 to G2T4N1. All patients with gNET‐G2 were included in this stage (*p* = 0.898) (Figure 2C). Patients with G2T3N1, G2T4N0, or G2T4N1 had relatively poor prognoses (Figure 2C) and they were classified as stage IIB and the rest as stage IIA. However, these two survival curves (*p* = 0.258) indicated that gNET‐G2 should not be divided into stage IIA and stage IIB (Figure 2D). Therefore, gNET‐G2 cases were classified as stage II without further subdivision.

#### 3.2.3 Stage III and IV

Before restaging gNET‐G3 and gNEC patients, we investigated whether patients with gNET‐G2 and gNET‐G3 could be combined into the same stage. The survival curves of patients with gNET‐G2 or G3 indicated that compared with gNET‐G2, gNET‐G3 had worse prognosis (gNET‐G2 served as the reference; gNET‐G3 HR, 4.180; *p* < 0.001) (Figure 2E and Figure S2B); suggesting that patients with NET‐G2 and NET‐G3 should not be integrated into the same group.

The survival curves of gNET‐G3 and gNEC (grouped into G4) were represented by a total of 13 curves (including metastases, defined as M1) (Figure 2F). G1M1 patients had good prognosis, with a 5‐year OS rate of almost 100%, similar to gNET‐G1. The survival curves of G4T1N0 and G4T2N0 were close to each other (*p* > 0.05) and were classified as stage IIIA. gNET‐G3 was classified as stage IIIB because it had poorer prognosis than stage IIIA. The survival curves of patients with G2M1, G4T1N1, G4T2N1, G4T3N0, and G4T3N1 were very close (*p* > 0.05) and therefore classified as stage IIIC. G3M1, G4T4N0, G4T4N1, and G4M1 almost overlapped (*p* > 0.05) and were classified as stage IV. The 3‐ and 5‐year OS rates of all patients in each stage are listed in Table 3 and Table 4. The novel TNMG staging system is presented in Table 5.

**TABLE 3:** The patients no. and OS of the TNMG stage in the SEER dataset

Variables	TNMG stage of the SEER dataset*					
	I (*n* = 924)	II (*n* = 189)	IIIA (*n* = 465)	IIIB (*n* = 30)	IIIC (*n* = 166)	IV (*n* = 471)
3‐year OS rate (%)	100	89.5	79.7	61.	46.5	9.5
5‐year OS rate (%)	99.2	84.2	71.6	51.3	35.8	6.8
Median OS (months)	>110	>104	122	62	31	6
HR (95% CI)	Reference	5.4 (2.9–10.0)	12.8 (8.0–20.5)	22.1 (10.8–45.1)	35.1 (21.7–56.6)	108.9 (69.3–171.2)

Abbreviations: CI, confidence interval; HR, hazard ratio; *n*, number of cases; Nx, any N stage; OS, overall survival; SEER, Surveillance, Epidemiology, and End Result; TNMG, T stage, N stage, M stage and G grade; Tx, any T stage.

^a^ Stage I comprised of G1T1N0 (*n* = 731), G1T2N0 (*n* = 126), G1T3N0 (*n* = 14), G1T4N0 (*n* = 5), G1T1N1 (*n* = 10), G1T2N1 (*n* = 11), G1T3N1 (*n* = 8), G1T4N1 (*n* = 2), G1M1 (*n* = 17); Stage II comprised of G2T1N0 (*n* = 138), G2T2N0 (*n* = 37), G2T3N0 (*n* = 3), G2T4N0 (*n* = 3), G2T1N1 (*n* = 2), G2T2N1 (*n* = 1), G2T3N1 (*n* = 4), G2T4N1 (*n* = 1); Stage IIIA comprised of G4T1N0 (*n* = 364), G4T2N0 (*n* = 101); Stage IIIB comprised of G3TxNx (*n* = 30); Stage IIIC comprised of G2M1TxNx (*n* = 11), G4T3N0 (*n* = 21), G4T1N1 (*n* = 36), G4T2N1 (*n* = 46), G4T3N1 (*n* = 52); Stage IV comprised of G3M1TxNx (*n* = 7), G4T4N0 (*n* = 20), G4T4N1 (*n* = 31), G4M1TxNx (*n* = 413).

**TABLE 4:** The patients no. and OS of the TNMG stage in the validation set

Variables	TNMG stage of the Chinese dataset*					
	I (*n* = 60)	II (*n* = 27)	IIIA (*n* = 7)	IIIB (*n* = 0)	IIIC (*n* = 82)	IV (*n* = 104)
3‐year OS rate (%)	100	87.5	66.7	NA	58.2	29.7
5‐year OS rate (%)	90.0	80.2	NA	NA	47.7	16.4
Median OS (months)	>114	>102	NA	NA	56	14
HR (95% CI)	Reference	9.2 (1.0–82.5)	23.4 (2.4–225.5)	NA	30.3 (4.1–221.7)	79.8 (11.0–574.9)

Abbreviations: CI, confidence interval; HR, hazard ratio; NA, not applicable; no., number; Nx, any N stage; OS, overall survival; SEER, Surveillance, Epidemiology, and End Result; TNMG, T stage, N stage, M stage and G grade; Tx, any T stage.

^a^ Stage I comprised of G1T1N0 (*n* = 50), G1T2N0 (*n* = 3), G1T3N0 (*n* = 0), G1T4N0 (*n* = 1), G1T1N1 (*n* = 0), G1T2N1 (*n* = 2), G1T3N1 (*n* = 1), G1T4N1 (*n* = 0), G1M1 (*n* = 3); Stage II comprised of G2T1N0 (*n* = 15), G2T2N0 (*n* = 5), G2T3N0 (*n* = 1), G2T4N0 (*n* = 0), G2T1N1 (*n* = 1), G2T2N1 (*n* = 3), G2T3N1 (*n* = 1), G2T4N1 (*n* = 1); Stage IIIA comprised of G4T1N0 (*n* = 2), G4T2N0 (*n* = 5); Stage IIIB comprised of G3TxNx (*n* = 0); Stage IIIC comprised of G2M1 (*n* = 16), G4T3N0 (*n* = 16), G4T1N1 (*n* = 4), G4T2N1 (*n* = 9), G4T3N1 (*n* = 37); Stage IV comprised of G3M1 (*n* = 2), G4T4N0 (*n* = 4), G4T4N1 (*n* = 34), G4M1 (*n* = 64).

**TABLE 5:** The proposed TNMG staging system for gNENs

Stage	TNMG substage			
	T	N	M	G
I	Any	Any	Any	1
II	Any	Any	0	2
IIIA	1–2	0	0	4
IIIB	Any	Any	0	3
IIIC	Any	Any	1	2
	1–2	1	0	4
	3	0–1	0	4
IV	4	0–1	0	4
	Any	Any	1	3–4

### 3.3 TNMG and TNM staging system in the SEER and validation dataset

**FIGURE 3:**
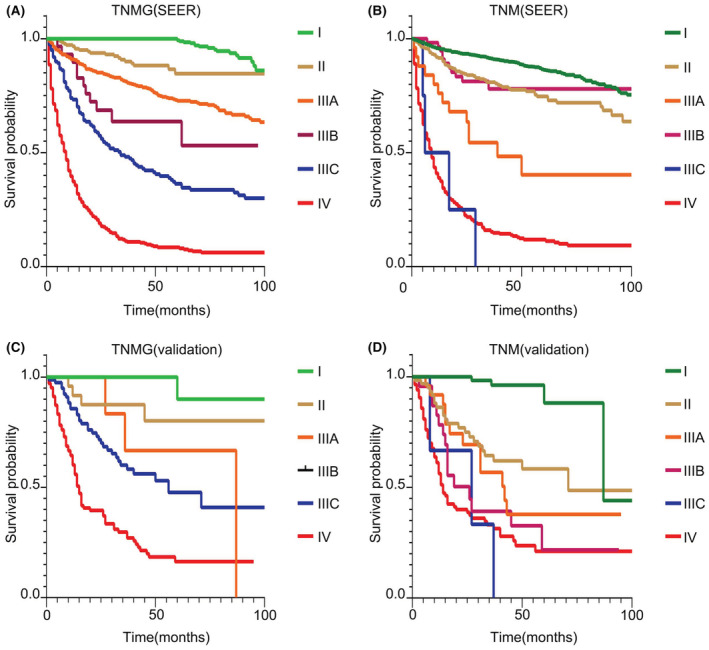
gNENs patients' prognostic stratification of TNMG staging system and TNM staging system in the SEER dataset and validation set. The prognostic curves of TNMG and TNM in SEER and validation set. The prognostic curves of TNMG in SEER (A) and TNM in SEER (B). the prognostic curves of TNMG in validation set (C) and TNM in validation set (D). SEER, Surveillance, Epidemiology, and End Result; TNM, tumor–node–metastasis; TNMG, T stage, N stage, M stage and G grade.

In the SEER dataset, a clear distinction between the survival curves of the TNMG staging system could be observed (*p <* 0.05), while for the 8th AJCC TNM staging system, no significant difference in prognosis between stage II and IIIB, and stage IIIC and IV could be observed. Further, the prognosis of stage IIIA patients was poorer than those of stage IIIB; confirming that the 8th AJCC TNM staging system could not optimally stratify patients with gNENs (Figure 3A,B).

In the validation dataset, except for stage II and IIIA, a clear distinction between the survival curves of the TNMG staging system could be observed (*p <* 0.05). For stage II and IIIA, a trend showing the prognosis of stage IIIA was poorer than stage II patients was observed, though the difference was not significant (*p* > 0.05). Additionally, all gNET‐G3 patients (*n* = 2) had distant metastasis and were classified as M1; thus, the survival curve of gNET‐G3 (stage IIIB) was not presented (Figure 3C). In the 8th AJCC TNM staging system, except for stage I, there was no significant difference in prognosis between stage II, III and IV patients (Figure 3D). In summary, in both the training and validation datasets, the TNMG staging system was more accurate in stratifying the OS of gNENs patients than the 8th AJCC TNM staging system.

### 3.4 Predictive accuracy and clinical performing ability of the TNMG stage

**FIGURE 4:**
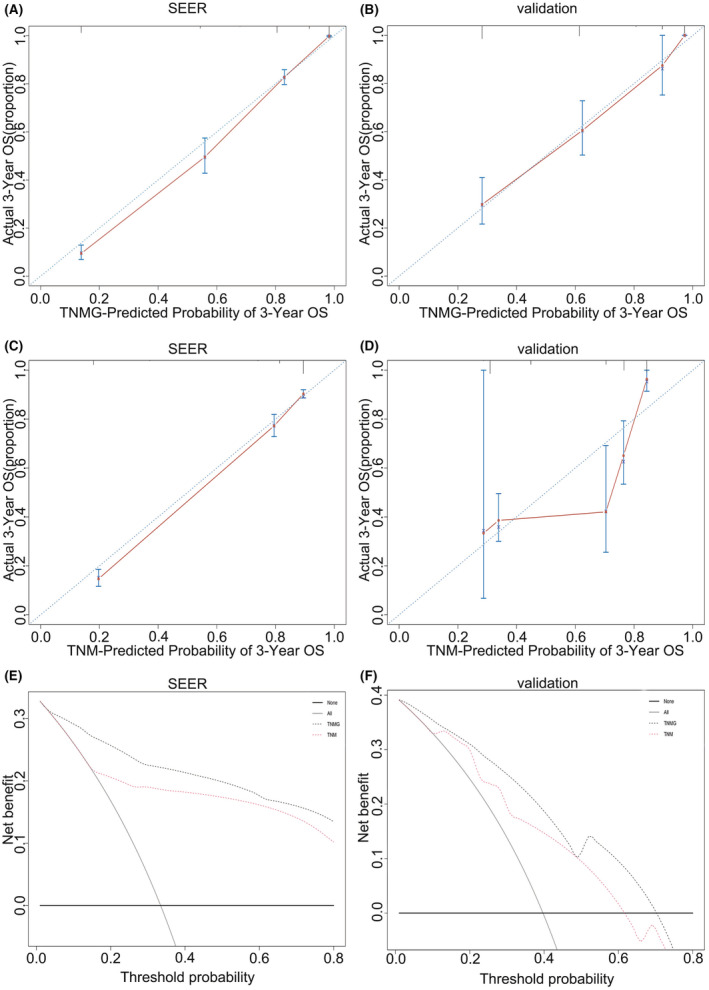
Calibration curves and DCA curves of 3‐year OS. The 3‐year OS Calibration curves of TNMG in SEER (A) and validation set (B). 3‐year OS Calibration curves of TNM in SEER (C) and validation set (D). 3‐year OS DCA curves in SEER series (E) and validation set (F). Clinical usefulness of different staging systems in predicting overall survival at various time points. The y axis represents net benefit. The *x*‐axis shows the threshold probability. The black dotted line displays the benefit of the novel staging system of TNMG. The red dotted line displays the benefit of the AJCC TNM stage. The gray line suggests that all patients have a poor prognosis, while the black line indicates that no patient has a poor prognosis. DCA, decision curve analysis; OS, overall survival; SEER, Surveillance, Epidemiology, and End Result; TNM, tumor–node–metastasis; TNMG, T stage, N stage, M stage and G grade.

**FIGURE 5:**
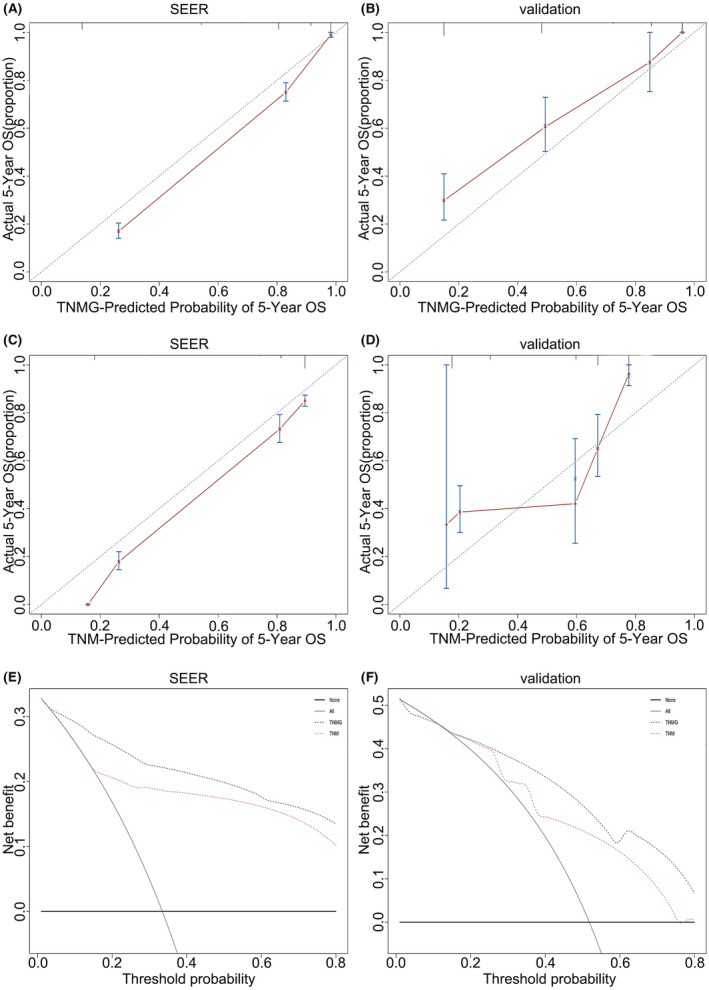
Calibration curves and DCA curves of 5‐year OS. Calibration curves and DCA curves. 5‐year OS Calibration curves of TNMG in SEER (A) and validation set (B). 5‐year OS Calibration curves of TNM in SEER (C) and validation set (D). 5‐year OS DCA curves in SEER series (E) and validation set (F). Clinical usefulness of different staging systems in predicting overall survival at various time points. The *y* axis represents net benefit. The x axis shows threshold probability. The black dotted line displays the benefit of the novel staging system of TNMG. The red dotted line displays the benefit of AJCC TNM stage. The gray line suggests that all patients have poor prognosis, while the black line indicates that no patient has poor prognosis. DCA, decision curve analysis; OS, overall survival; SEER, Surveillance, Epidemiology, and End Result; TNM, tumor–node–metastasis; TNMG, T stage, N stage, M stage and G grade.

The TNMG staging system had better C‐index in predicting OS than the 8th AJCC TNM staging system (0.79, 95% CI: 0.77–0.81) in both the SEER (0.87, 95% CI: 0.86–0.88) and validation (0.77; 95% CI: 0.73–0.80 vs. 0.75; 95% CI: 0.71–0.79) datasets. The AUC of the 3‐ and 5‐year OS for the TNMG staging system in the SEER and validation dataset were higher than the 8th AJCC TNM staging system (Table 6). In addition, the calibration curve (3‐year OS) of the TNMG indicated its predicted survival rate was closer to the corresponding actual survival rate, both in the SEER and validation datasets (Figure 4A,B), while the calibration of the TNM showed relatively poorer consistency (Figure 4C,D). The 3‐year OS of DCA revealed the TNMG staging system had a superior net prognostic benefit in both the SEER and validation set, compared with the 8th AJCC TNM staging system (Figure 4E,F). The 5‐year OS calibration curves and DCA curves indicated that the TNMG staging system was more accurate in prognosticating the 5‐year OS rate than the 8th AJCC TNM staging system (Figure 5).

**TABLE 6:** C‐index and AUC in the SEER and validation datasets

Variables	Datasets			
	SEER		Validation	
	TNMG	TNM	TNMG	TNM
C‐index	0.87 (95%CI: 0.86–0.88)	0.79 (95%CI: 0.77–0.81)	0.77 (95%CI:0.73–0.80)	0.75 (95%CI: 0.71–0.79)
AUC (3‐year OS)	0.936	0.843	0.817	0.779
AUC (5‐year OS)	0.910	0.806	0.818	0.790

Abbreviations: AUC, areas under the receiver operating characteristic curve; CI, confidence interval; C‐index, Harrell's c‐statistics; SEER, Surveillance, Epidemiology, and End Result; TNM, tumor–node–metastasis; TNMG, T stage, N stage, M stage and G grade.

## 4 DISCUSSION

In this study, we established a novel staging system by incorporating the WHO G grade into the 8th AJCC TNM stage system based on the training data of the SEER database, which was then validated in a two‐center Chinese patient cohort. This novel staging system was termed the TNMG staging system. It demonstrated superior performance than the 8th AJCC TNM staging system for predicting the OS of gNENs patients.

Although the TNM stage has an important role in standardizing the survival prognosticating of gNENs patients, however, predicting the prognosis of these patients solely relying on the TNM stage could be inaccurate as it ignored grade‐related factors such as Ki‐67 and mitotic count, which are the basis for defining the WHO G grade and was proved to have an obvious improvement in prognosis estimation; particular in neuroendocrine neoplasms.^5^, ^21^, ^22^, ^23^, ^24^ This study also revealed the limitation of the 8th AJCC TNM staging system when applied in gNETs as the prognosis of different G grades of the same TNM stage was statistically different (Figure 1). Importantly, we showed that the G grade weighed a lot in predicting prognosis. The HR value of G grade was 2.270, larger than any other prognostic factors in multivariate survival analyses, except for the M stage. Robert et. al analyzed 6747 pNETs cases and proposed a staging system showing that high grade (III/IV) pNET cases had poorer prognosis than low/mediate grade (I/II) patients (HR = 2.2); implying G grade as a strong predictive indicator for OS.^25^ Moreover, a multicenter retrospective analysis on metastatic and locally advanced pNECs indicated that G grade was also an important risk factor for disease progression.^26^ Another comprehensive analysis from a large Chinese institution for pNENs concluded that the 2017 WHO grading classification for p‐NENs could successfully allocate patients into four groups with distinct clinical features and survival differences.^27^ An increasing number of studies indicated that the G grade played a decisive role in predicting the prognosis of gastroenteropancreatic neuroendocrine neoplasms (GEP‐NENs).^25^, ^26^, ^27^, ^28^, ^29^, ^30^, ^31^ To date, a novel staging system for pNENs has proposed that G grade should be taken into account, together with TNM stage, and the new staging system showed better predicting ability than the 8th AJCC TNM staging system,^14^ but a similar model for gNENs was yet to be investigated. Here, we constructed the first staging system incorporating the T stage, N stage, M stage, and G grade for gNENs.

Different from the 8th AJCC staging system, TNMG stage I contained all patients with gNET‐G1 (including M1 cases). In the SEER dataset, patients with TNMG stage I had very good prognoses, with 5‐year OS of ~100%. Tsolakis et al. performed a meta‐analysis for gastric neuroendocrine neoplasms type 1 (gNEN1), and found that the metastatic propensity of GNEN1 was low and the overall prognosis of gNET‐G1 was excellent, with a 5‐year DSS of 100%.^32^ Patients with gNET‐G2 (excluding M1 cases) were distributed into the TNMG stage II and had a relatively poorer 5‐year OS, approximately 84.2%. We further tried to divide stage II into IIA and IIB, and despite observing a trend suggesting patients with stage IIB manifested poorer prognosis than those of stage IIA, the difference was not statistically significant (*p* = 0.258). Thus, a larger cohort data study should be performed to validate this finding.

Previously, all NET‐G3 neuroendocrine samples, or high‐grade NENs, were considered as NEC and were uniformly evaluated and treated.^33^, ^34^, ^35^ Subsequently, the classification based on a consensus conference held at the International Agency for Research on Cancer (IARC) in November 2017 separately distinguished pancreatic well‐differentiated NE tumors (Pan‐NETs) and poorly differentiated NE carcinomas (Pan‐NECs) morphologically.^36^ Currently, the 2019 WHO classification of tumors of the digestive system generated it to the gastrointestinal tract and hepatopancreatobiliary organs. NET‐G3 shows well‐differentiated morphology and NEC poorly differentiated morphology, but both belong to high‐grade NENs.^5^ This study found that whether in the SEER (*n* = 37/2245; 1.65%) or validation (*n* = 2/280; 0.71%) dataset, there was only a small percentage of patients diagnosed as gNET‐G3, with 37 patients in the SEER dataset and 2 patients in the validation set. High evidence level data of NET‐G3 remain scarce because most data were from retrospective studies, and were often from reassessment and reclassification of NEC samples.^37^, ^38^ More and more studies have confirmed that well‐differentiated NET‐G3 has a better prognosis than NEC because of distinct tumor characteristics and such assessments are important for a personalized treatment approach.^37^, ^39^, ^40^ However, in the SEER dataset, we found that patients with gNET‐G3 (excluding M1 cases) had worse prognoses than patients diagnosed as T1N0 and T2N0 gNEC (5‐year OS:51.3% vs. 71.3%, *p* = 0.042) and should therefore be further validated in larger datasets.

TNMG stage IV comprised of G3M1, G4M1, G4T4N0, and G4T4N1. In this stage, we observed that the prognosis of gNET‐G3 and gNEC were similar when distant metastases occurred. As shown in Figure 2F, the survival curves of G3M1 and G4M1 overlapped (*p* = 0.639). Unexpectedly, patients with G4T4N0 and G4T4N1 had similar prognosis as G3M1/G4M1 (*p* = 0.309). This outcome implied that when gNEC invaded the serosal membrane or adjacent structures, the prognosis was comparable to gNEC with distant metastasis, regardless of whether there was lymph node metastasis or not. Additionally, we found that the prognosis of gNEC patients with TNM stage IIIC was not statistically different compared with TNM stage IV in both the training and validation datasets. The prognostic curves of the TNM stage IIIC intersected with that of TNM stage IV (*p* > 0.05) (Figure 3B,D). A modified staging system for gNEC based on the AJCC and European Neuroendocrine Tumor Society (ENETS) system demonstrated that gNEC patients with TNM stage IIIC disease had a similar prognosis to those with stage IV disease.^15^ Wang et al. proposed a new staging system for pNENs combining the TNM stage and G grade, which showed better predicting ability than the 8th AJCC TNM staging system. However, in their study, they just combined the TNM stage (I, II, III, IV) and G grade (G1, G2, G3), rather than considering every subgroup in the TNM stage. For example, TNM stage I contained three subgroups including T1N0, T1N1, and T2N0. All these subgroups should be recombined with G grade when establishing the new staging system. Another issue was that they did not distinguish pNET‐G3 and pNEC, which are categorized as G3 in this study.^14^ Jackson et al. established a staging system incorporating the TNM stage and G grade for neuroendocrine tumors of the lung and it was also proved to be more accurate than the 8th AJCC TNM staging system. In their staging system, all patients with NET‐G1 were included in stage I and all patients with NET‐G2 were allocated to stage II, which was concordant with this present study findings. Though they have taken every subgroup of the TNM stage into consideration, they did not make further distinction between NET‐G3 or NEC, similar to a study by Wang et al.^14^ Besides, they did not analyze patients with stage IV.^41^ In this present study, we not only elaborately analyzed every subgroup of the TNM stage combined with G grade, but also differentiated between gNET‐G3 (G3) and gNEC (G4).

There were some important limitations of this study worth mentioning. First, it was limited by its retrospective nature. Second, a limited number of patients were included in the validation set. Third, this validation set is a two‐center database from the Chinese southern province and might not represent all gNENs patients in China. Thus, larger multicenter studies should be performed for further validation.

In conclusion, a novel staging system for gastric neuroendocrine neoplasms is proposed in this study. It could predict the 3‐ and 5‐year OS rates of gNENs patients more accurately than the 8th AJCC TNM staging system.

## AUTHOR CONTRIBUTIONS

**Rihong Zhang:** Data curation (lead); formal analysis (lead); investigation (equal); resources (lead); software (lead); validation (lead); writing – original draft (lead). **Yu Guo:** Data curation (equal); formal analysis (equal); investigation (equal); resources (equal); validation (equal). **Youliang Wang:** Data curation (equal); formal analysis (equal); resources (equal); software (equal). **Li Hu:** Formal analysis (equal); resources (equal); software (equal). **Cheng Fang:** Data curation (equal); funding acquisition (supporting); resources (equal). **Yujie Yang:** Data curation (equal); resources (equal). **Xianqi Yang:** Data curation (equal); resources (equal). **Luohai Chen:** Data curation (equal); resources (equal). **Jie Chen:** Conceptualization (equal); writing – review and editing (equal). **Wei Wang:** Funding acquisition (supporting); project administration (equal); supervision (equal); validation (equal); writing – review and editing (equal). **Xiaowei Sun:** Data curation (lead); project administration (lead); supervision (lead); validation (lead); writing – review and editing (lead).

## FUNDING INFORMATION

This study was supported by the Natural Science Foundation of Guangdong Province (Grant No. 2114050002182), Young Teacher Training Program of Sun Yat‐sen University (Grant No. 19ykpy172), National Natural Science Foundation of China (Grant No. 81802451), and Natural Science Foundation of Guangdong Province (Grant No. 2018A030313827).

## CONFLICTS OF INTEREST

The authors declare no competing or financial interests.

## Supporting information


Figure S1

## Data Availability

The key raw data have been deposited into the Research Data Deposit (http://www.researchdata.org.cn), with the Approval Number of RDDA2022833813 and the datasets used in this study are publicly available.
