# Supplementary material for: Novel staging for gastric neuroendocrine neoplasms by incorporating the WHO grading into the TNM staging system
Source: Cancer Med. 2022 Nov 16;12(6):6623–36. doi: 10.1002/cam4.5437 (PMC10067116; doi:10.1002/cam4.5437)
Supplement: Supplementary file 1 — Figure S1 [file CAM4-12-6623-s001.docx]

**Figure S1. A flow diagram of the selection process for patients with gastric neuroendocrine neoplasms in the SEER dataset.**

5874 patients in the SEER database was inspected pathologically diagnosed as gNENs and

treated from 2004 to 2018. Finally,2245 patients are selected for analysis after screening.
